# Ecology and Evolution in an Open World (or: why supplementary data are evil)

**DOI:** 10.1002/ece3.2101

**Published:** 2016-03-28

**Authors:** Allen J. Moore, Andrew Beckerman

**Affiliations:** ^1^ Department of GeneticsUniversity of GeorgiaAthensGA30602USA; ^2^ Animal & Plant SciencesUniversity of SheffieldSheffieldS10 2TNUK

It has been too long since we have provided an editorial overview – we hope you have not missed us. However, let us catch you up on the journal and our continued focus on providing an outlet for research in ecology and evolution.

First, we can celebrate the growth and vigor of our journal. Our partnership with society‐based (BES, ESEB, SSE – and if you do not know these acronyms, you should find out. Best societies ever. Really) and Wiley journals remains strong, and we continue to receive excellent manuscripts transferred with reviews from our partners. Space limitations and the weight of submissions mean that all of our partners receive many more manuscripts than they can publish, and most of these will eventually find an outlet. The rigorous review process orchestrated by our partners improves all of the papers submitted to their journals, and if a paper is rejected, the review may have been for naught. By offering to transfer a paper with these reviews, our partners can provide yet another service to the authors that submit their work to them, the opportunity to be considered for publication by us (typically) without the potential double jeopardy of a second round of reviews. Moreover, it helps the reviewer by ensuring that her/his work has an influence. Thus, transferring papers with reviews benefits authors and reviewers and helps reduce the growing burden on the reviewing community.

The option to transfer a paper is just that – an option. The decision to cascade their work resides solely with the authors. Authors often choose to pursue publication elsewhere, but many take the option to transfer. Transferred papers have been carefully reviewed. Indeed, often the papers come to us having already been reviewed twice (once after revision) at the original journal. Our goal was to treat these papers with respect for the authors and referees, both of whom have invested time, and as rapidly as possible. After all, none that are transferred were considered flawed (we ask that the editors of our partner journals only transfer papers that should be published after some editing or attention). The most common reason for rejection we see in papers that are transferred is “lacking in novelty,” which is an opinion (rarely shared by the authors) and readily fixed. Our goal was to provide a high profile, open, outlet that puts your research into the hands of the community as quickly as possible.

What, then, do we do with papers that are transferred? We are guided by our philosophy of being “author friendly”. In its simplest form, this philosophy means that we approach every manuscript asking the question of “let us find a way to publish these data and ideas” rather than “what is wrong with this paper?” This does not mean we accept whatever you write, and we (the editors) try to work with the authors to ensure that they are presenting their work with a good story and a reasonable tone and approach. Nevertheless, “overinterpreted” can be in the eyes of the reader and when in doubt we are happy to let the community decide on the fate of a paper.

This has led to an interesting phenomenon: The direct submissions to Ecology and Evolution are rapidly increasing. While we are delighted by the positive response of the community to the journal, and by the diversity of papers we receive that strengthens Ecology and Evolution as a research outlet for everyone, this presents us with a dilemma. Where we strive for a one‐week turnaround with transferred manuscripts, direct submissions (and manuscripts transferred without reviews – those editorially rejected for being out of scope, etc.) end up allocated to the workflow any “normal” manuscript goes through at any journal. We retain our author‐centered philosophy here and certainly try to provide a rapid decision, but the paper has to be assigned to an associate editor, reviewers found, reviewers agree, and reviews returned before a decision can be made. Thus, for direct submissions Ecology and Evolution is acting as a traditional open‐access journal.

The full range of decisions occurs for direct submissions, from “reject” to “accept with minor revisions” (has anyone in history ever received “this is perfect” on a first submission? No? We have not either). We suspect we are no faster than any other journal with direct submissions, given many of the steps are out of our control. Moreover, the rapid growth creates teething pains for figuring out who should handle manuscripts, and we apologize if yours has been caught clogged in the system for too long. We are investigating how we might become more efficient because we too worry that the process just should not take so long. The good news, once a paper is accepted it will appear online very rapidly (dependent in large part on the speed with which authors deal with page proofs). But whatever we decide, we will continue to promote our “author‐friendly” philosophy. After all, we too are active researchers and authors, and we also check daily on the status of our manuscripts submitted to other journals.

This brings us to the second topic and the reason for the title. We are an open‐access journal, an online‐only publication, and this brings authors and publishers a lot of advantages. Not only will we (through Wiley) continue to investigate ways that online publishing can enhance your work and facilitate reaching your audience, there are many areas you can use today. Color is encouraged in your graphs – the use of black and white really reflects the approach to publishing from the last century. Include photographs (we already encourage at least one), color graphics, maps, and contours with color – whatever enhances your ability to communicate your message. Be creative.

There is another advantage to online and open publication. We really cannot fathom why supplementary material continues in its present form. We already promote data sharing through open archives and are actively discouraging supplementary material. Regardless of the approach of other journals, unless you have a video, or perhaps a huge dataset (which really should be in a data archive anyway), there should not be supplementary material in an online article. Supp Mat, as it is not fondly known (usually proceeded by an impolite word from at least one of us), was invented by glossies that had too few pages to actually provide accurate information. Okay, that may be harsh and inaccurate and is just an opinion. But why in the world would you bury any information that is helpful or even necessary to understand your work? We think such information is better placed in an appendix that forms part of the paper. There is no overhead here, as there are no page limits with an online‐only journal. This would make sure that ALL of the information needed to understand your work is provided in a single download. If you have additional helpful figures, put them in an appendix. If you have detailed methods, or code, or tables with the original sources for a meta‐analysis, and appendix is fine. Use the flexibility of online publishing to be accurate and complete.

Supplementary material is currently where data and methods go to die, never to be viewed again. We are author friendly. We also strive to be reader friendly. Our readers are busy, and while it may seem trivial to download two files instead of one, how do you ensure that the two remain linked in whatever folder in which you downloaded files? If you are like us, two months later you are staring at an unhelpfully labeled “smith_et_al_supp_mat” file wondering just what might be in it and which Smith this might be and why we thought her paper so memorable. So, whatever the rationale or rules at other journals, let us be open and share our work. Make use of appendices instead of sup mat. Create a single file. Use visuals where possible. Upload your data to GenBank or Dryad or wherever you make it freely available. Be reader friendly. We will continue to work to produce a journal that is author friendly as well.

